# Multimodal Super‐Resolution Imaging of Nitrogen‐Vacancy Centers via High‐Index‐Induced Structured Illumination Microscopy and Optically Detected Magnetic Resonance Spectrometry

**DOI:** 10.1002/advs.202522495

**Published:** 2026-03-03

**Authors:** Kyu Ri Choi, Mohammed Zia Jalaludeen, Samuel Begumya, Yan Qiu Du, Dong Hee Park, Bin Chan Joo, Jae Hwan Yoo, Síle Nic Chormaic, Shilong Li, Yeon Ui Lee

**Affiliations:** ^1^ Department of Physics Chungbuk National University Cheongju Chungbuk Republic of Korea; ^2^ Light‐Matter Interactions for Quantum Technologies Unit Okinawa Institute of Science and Technology Graduate University Onna Okinawa Japan; ^3^ School of Electronics and Information Engineering Heilongjiang University of Science and Technology Harbin China; ^4^ College of Information Science and Electronic Engineering Zhejiang University Hangzhou China

**Keywords:** nitrogen‐vacancy centers in diamond, optically detected magnetic resonance, structured illumination microscopy, super‐resolution imaging

## Abstract

Structured illumination microscopy (SIM) allows for optical imaging with spatial resolution that surpasses the diffraction limit. To further enhance this super‐resolution capability, we present a multimodal approach that integrates SIM with optically detected magnetic resonance (ODMR) of nitrogen‐vacancy (NV) centers in diamond—a platform considered one of the most promising platforms for quantum sensing and computing. Both theoretical modeling and experimental results reveal that diamond's high refractive index supports finer structured illumination patterns, thereby accessing higher spatial frequencies and yielding improved resolution. This combined strategy not only enhances NV center localization precision and achieves sub‐100‐nm resolution but also facilitates spin coherence analysis. Leveraging the high refractive index of diamond in SIM thus offers a powerful pathway toward advancing quantum applications at the nanoscale.

## Graphical abstract

This work demonstrates a multimodal super‐resolution imaging technique for nitrogen‐vacancy centers by integrating high‐index‐induced structured illumination with optically detected magnetic resonance. By utilizing diamond's high refractive index, the method achieves sub‐100‐nm spatial resolution and enhanced localization. This dual‐modulation strategy enables simultaneous high‐fidelity spatial imaging and spin coherence analysis, providing a robust tool for advancing nanoscale quantum sensing.

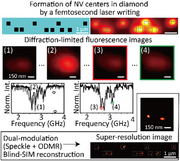

## 1 Introduction

Super‐resolution imaging techniques have revolutionized optical microscopy by surpassing the diffraction limit, enabling nanoscale visualization of biological and quantum systems [1, 2, 3, 4, 5, 6]. Among the many systems benefiting from this advancement, nitrogen‐vacancy (NV) centers in diamond have attracted increasing attention in the field of super‐resolution imaging due to their unique combination of optical and quantum properties [7, 8, 9]. NV centers serve as powerful probes for both live‐cell imaging and quantum sensing, offering photostability, long spin coherence time, and optically addressable spin states [10, 11, 12, 13, 14, 15]. To fully exploit these properties, wide‐field super‐resolution imaging of NV centers is essential, especially for applications in high‐speed quantum information processing and high‐throughput biosensing. Methods such as single‐molecule localization microscopy (SMLM) and stimulated emission depletion (STED) microscopy offer exceptional spatial resolution but often require scanning‐based acquisition or point‐by‐point excitation of specific fluorophores with high‐power illumination, making wide‐field imaging challenging or inefficient. Structured illumination microscopy (SIM), on the other hand, provides a relatively simple and efficient approach for super‐resolution imaging across large fields of view, while maintaining lower phototoxicity and compatibility with a broader range of samples [16, 17]. However, conventional linear implementations of SIM typically yields only a twofold resolution improvement [18, 19]. Although nonlinear SIM variants, such as saturated and photoactivated SIM, can in principle achieve higher resolution gains, they generally require high excitation intensities and specialized photoswitchable fluorophores, which are not naturally compatible with dense NV ensembles in bulk diamond and simultaneous quantum sensing measurements.

To overcome the resolution limit of linear SIM, various high‐refractive‐index substrates have been explored, such as composite metamaterials [20, 21], plasmonic structures [22, 23], conjugated polymer films [24, 25, 26, 27], epsilon‐near‐zero organic nanomembranes [28], and photonic chips [29, 30]. These approaches manipulate light propagation and structured illumination patterns to enhance spatial frequency transfer, leading to improved imaging resolution. Nonetheless, most of these methods require engineered substrates that are not inherently compatible with NV center imaging, which relies on diamond as the host material. Despite diamond's high refractive index (𝑛 ≈ 2.4), compared to typical immersion media used in SIM (e.g., water or oil), its potential role in enhancing SIM resolution for NV center imaging has not been explicitly demonstrated.

In this work, we combine theoretical modeling and experimental validation to demonstrate how the high refractive index of diamond enhances SIM imaging resolution of NV centers. By analyzing structured illumination pattern formation and spatial frequency transfer in a high‐index environment, we establish the fundamental advantage of diamond for super‐resolution imaging. Moreover, we integrate optically detected magnetic resonance (ODMR) spectrometry with SIM to achieve high‐resolution NV center imaging while simultaneously probing spin‐dependent fluorescence. Notably, we show that the frequency‐dependent fluorescence modulation intrinsic to ODMR can act as an additional structured excitation source, actively contributing to the SIM reconstruction process. This integrated approach enhances the spatial resolution by leveraging the high‐index environment to improve the formation of structured illumination patterns and by modulating fluorescence brightness in both the temporal and spatial domains. Such a dual‐modulation strategy—combining optical speckle structuring and microwave‐driven spin modulation—enables sub‐diffraction imaging with enhanced spatial precision. Our findings provide new insights into the role of high‐index materials in super‐resolution microscopy and position diamond as a powerful platform for advanced applications in quantum sensing and nanophotonic imaging.

## 2 Results

### 2.1 Laser writing of NV Centers in Diamond

**FIGURE 1:**
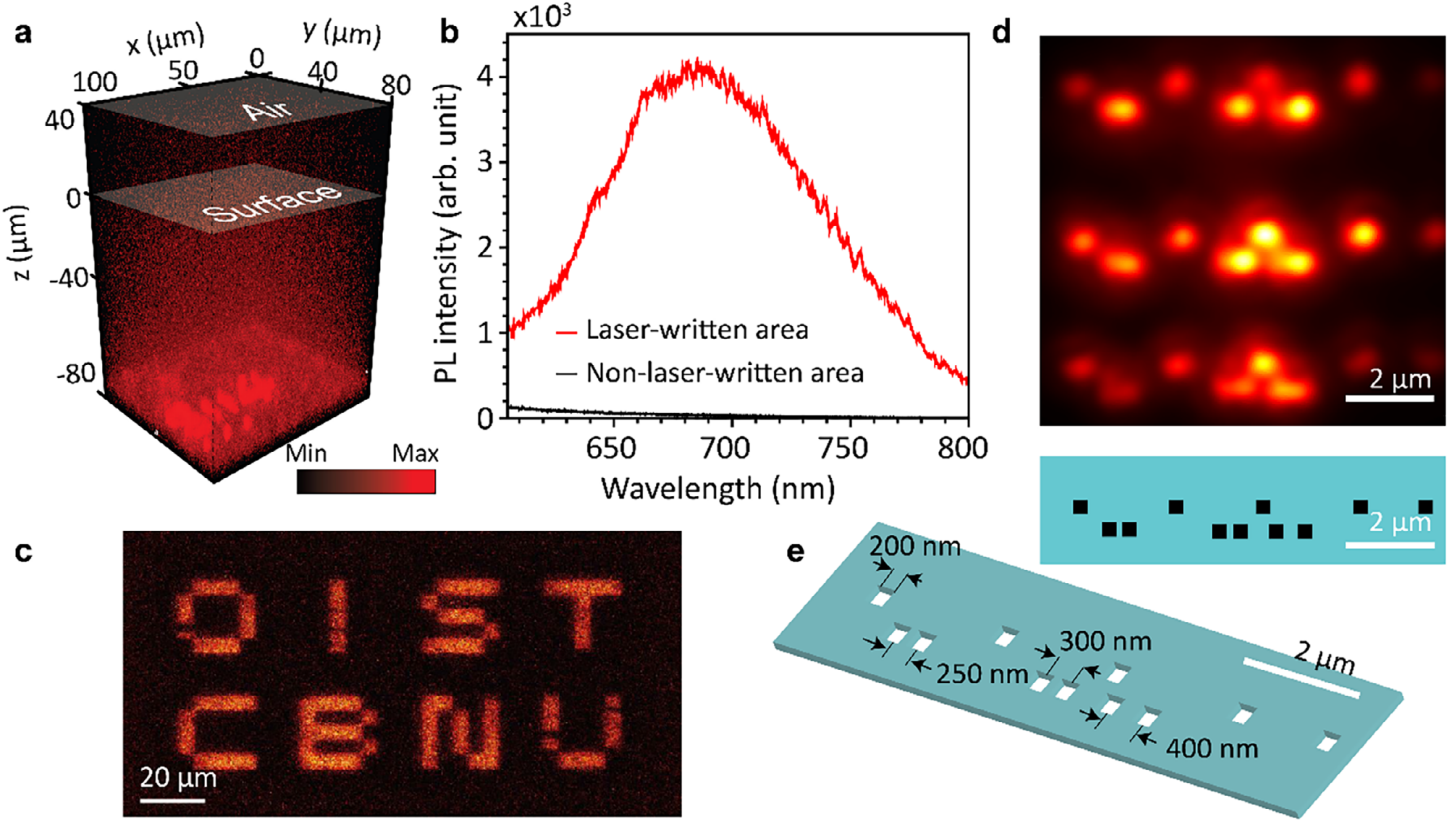
Characteristics of laser‐written NV centers in diamond. (a) *Z*‐stacked volumetric emission view of the diamond sample, where 0 µm (along *z*) denotes the diamond surface, and negative values correspond to depths inside the diamond. An interface layer with enhanced emission is observed at approximately 100 µm beneath the surface. The 514‐nm continuous‐wave excitation laser was focused through an objective lens (Plan‐Apochromat 40×, NA 0.95), and the resulting fluorescence signals (600–800 nm) were detected. (b) Comparison of photoluminescence (PL) intensity at the interface layer between the laser‐written and non‐laser‐written areas. (c–e) Images of laser‐written patterns at the interface layer, with scale bars of (c) 20 µm, (d) 2 µm, and (e) 2 µm, respectively.

The NV centers were created in a type 1b high‐pressure high‐temperature (HPHT) diamond (Element Six Technologies) by femtosecond laser irradiation at 780 nm, performed using a confocal microscope (Zeiss LSM 710) equipped with a tunable infrared pulsed laser (Coherent Chameleon Ultra II). The fabricated NV centers were localized within an interface layer approximately 100 µm beneath the diamond surface, as revealed by volumetric *Z*‐stack fluorescence imaging (Figure 1a). Notably, this laser‐based writing technique enables the direct and in situ formation of NV centers under ambient conditions, eliminating the need for high‐temperature annealing [31], simplifying the process, and minimizing collateral damage or graphitization [32, 33, 34, 35, 36]. Further details on the fabrication protocol and NV center characterization are provided in Figures S1 and S2.

A comparison between the laser‐written and non‐laser‐written areas, as shown in Figure 1b, reveals a significantly enhanced emission in the laser‐written area, indicating that femtosecond laser irradiation efficiently induces NV center formation. This enhancement is consistent with the generation of high‐density NV ensembles by femtosecond laser writing, as discussed in Figure S2. In this process, laser‐generated neutral vacancies (GR1 centers) recombine with nearby substitutional nitrogen atoms within the focal volume, leading to the formation of NV centers and the enhanced NV emission peak observed in Figure 1b. Using this laser writing technique, arbitrary patterns can be inscribed into the interface layer, as demonstrated in Figure 1c,d. In particular, a dot array pattern with dimensions of 200×200 nm^2^ was written into the interface layer (Figure 1d,e): the pattern consists of two lines of dots; the upper line contains single dots, while the lower line contains pairs of dots separated by 50 nm, 100 nm, and 200 nm, respectively. These pairs are arranged such that the shortest spacing appears on the left and the largest on the right. This patterned array was repeated multiple times across the interface layer. As shown in Figure 1d, dot pairs with smaller separations appear diffraction‐limited, indicating that their separation is below the resolution limit of the optical imaging system.

### 2.2 Generation of High‐Spatial‐Frequency Illumination Patterns in Diamond

**FIGURE 2:**
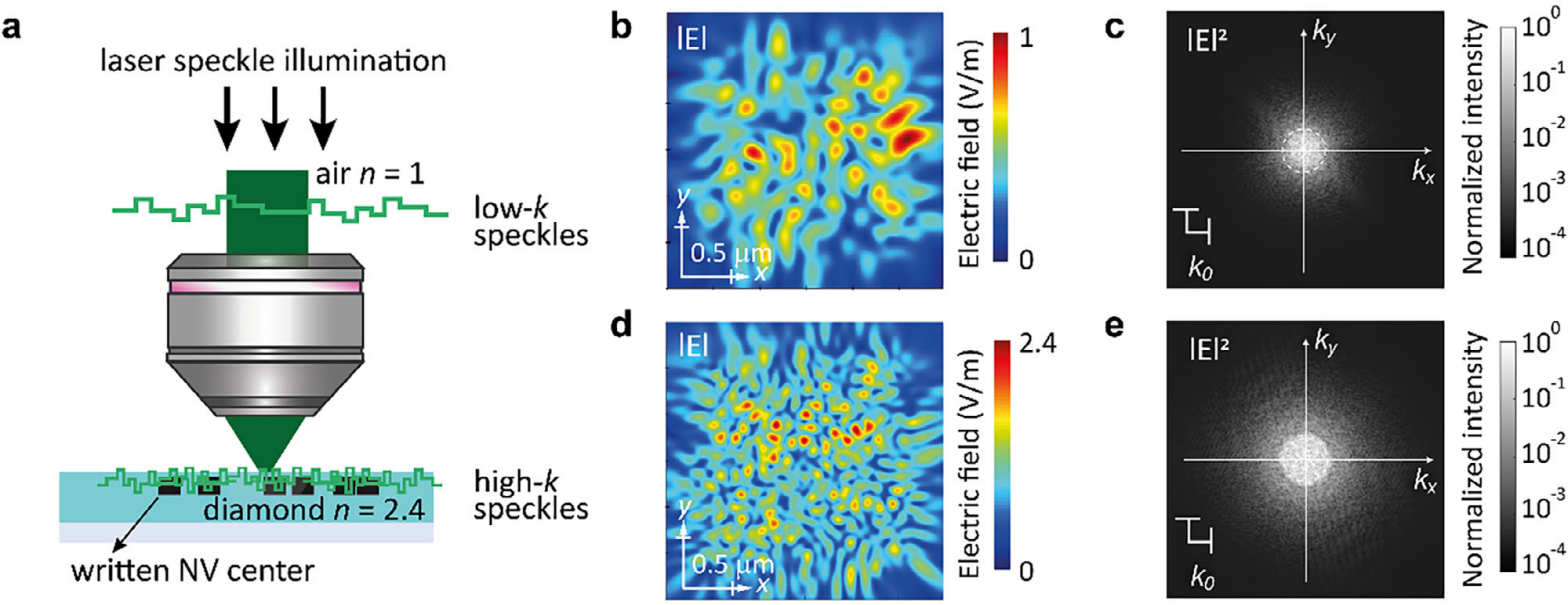
Generation of high‐*k* speckle patterns in a high‐index diamond medium. (a) Schematic of laser speckle compression by the diamond. The incident speckle patterns with low‐*k* in air are compressed upon entering the diamond due to its high refractive index (*n* = 2.4), resulting in speckle patterns with high‐*k*. (b) Electric field distributions of the speckle pattern in air calculated using FDTD, showing relatively coarse features. (c) Corresponding Fourier‐transformed intensity distribution, where the spatial frequency components are limited by the air cutoff wavenumber *k*_0_ = 2π/λ. (d) Electric field distributions of the speckle pattern in diamond, exhibiting finer and denser features compared to those in air. (e) Corresponding Fourier‐transformed intensity distribution revealing extended *k*‐space content with a larger *k*_cut‐off_ = *nk*_0_, thereby allowing access to higher spatial frequencies. This extension in *k*‐space support is essential for achieving sub‐diffraction‐limited resolution in structured illumination‐based super‐resolution microscopy.

To achieve super‐resolution imaging of the abovementioned sub‐diffraction‐limited NV centers in diamond, we employ a SIM scheme based on high‐spatial‐frequency (high‐*k*) speckle pattern modulation. As shown in Figure 2a, when random speckle illumination is introduced from air onto the diamond substrate, it encounters the high refractive index of diamond (𝑛 ≈ 2.4) [37], compared to that of air (𝑛 = 1). As a result, incident illumination patterns with relatively low spatial frequencies (low‐𝑘, diffraction‐limited) are compressed upon entering the diamond, transforming into high‐*k* components. Such high‐*k* illumination enables the resolution of fine structural details beyond the diffraction limit, providing deeper insight into the spatial characteristics of NV centers in diamond.

Finite‐difference time‐domain (FDTD) simulations were performed to better illustrate the illumination compression process. Figure 2b,c presents the speckle field distribution in air. The electric field map (Figure 2b) shows relatively coarse, diffraction‐limited patterns, and the corresponding spatial frequency spectrum (Figure 2c), obtained by Fourier transformation, is confined within the limited spatial bandwidth bounded by the free‐space spatial‐frequency *k*_0_. In contrast, the speckle patterns simulated inside the diamond (Figure 2d,e) exhibit denser and finer structural features in real space (Figure 2d), attributed to the increased transverse wavevector components allowed by the higher refractive index. The associated Fourier‐transformed intensity map (Figure 2e) shows a broadened spectral distribution with significant components beyond the free‐space cutoff *k*_0_, indicating the presence of high‐*k* content. Therefore, the high refractive index of diamond compresses incident speckle patterns into high‐*k* excitation fields, which modulate the fluorescence emission of NV centers at sub‐diffraction spatial scales. This capability facilitates structured illumination‐based super‐resolution imaging of NV centers in diamond, as demonstrated in the following.

### 2.3 Combining Super‐Resolution Microscopy and ODMR Spectrometry

**FIGURE 3:**
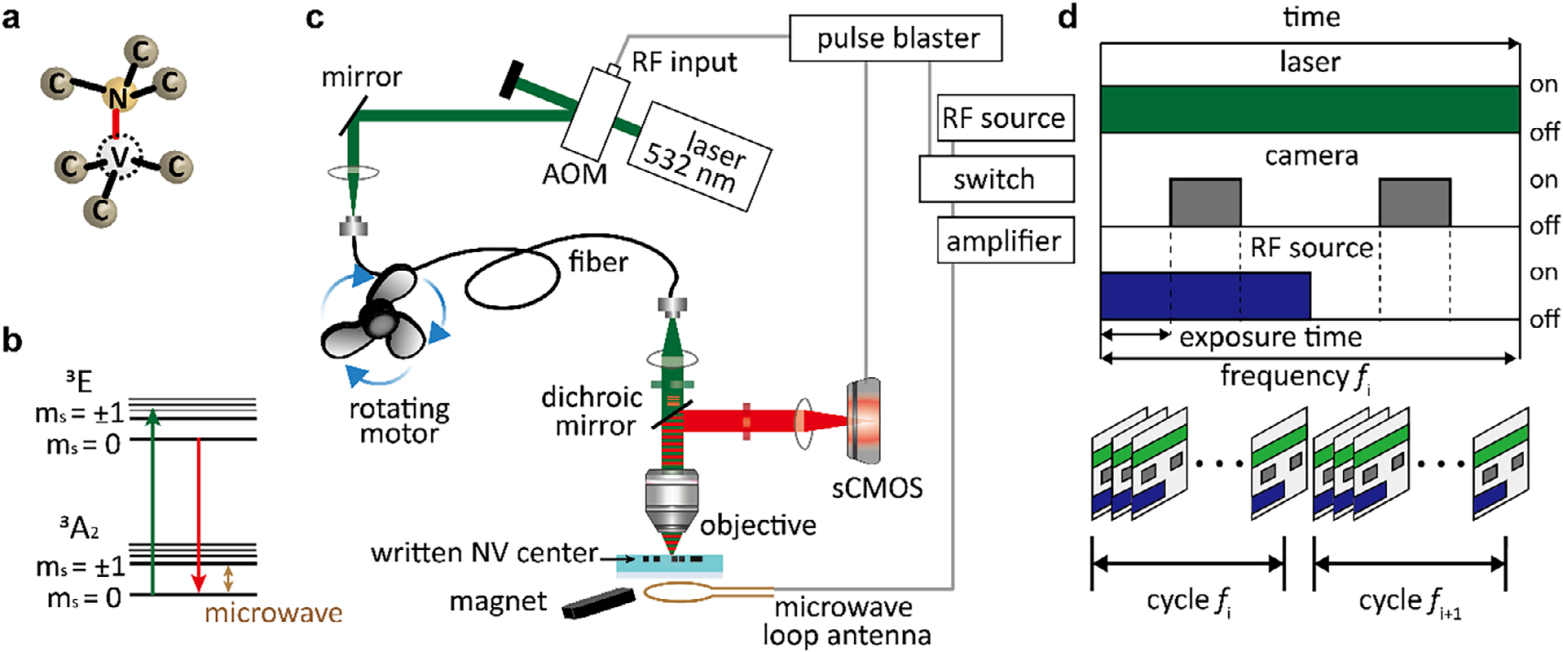
Structured illumination ODMR experimental setup and data acquisition procedure. (a) Structure of an NV center in diamond. (b) Simplified electronic energy level diagram of the NV center. (c) Schematic of the experimental setup. A 532‐nm continuous‐wave laser is modulated using an acousto‐optic modulator (AOM), and the first‐order diffracted beam is selected through a pinhole. The laser beam is then coupled into a mechanically deformed multimode optical fiber positioned near a rotating motor to generate dynamic speckle patterns. The resulting laser speckle is directed toward the diamond sample, which is also exposed to a static magnetic field from a magnet and a radio‐frequency (RF) electromagnetic field from an RF source via a loop antenna. Fluorescence emission from NV centers in the diamond sample is collected and imaged onto a scientific CMOS (sCMOS) camera. A pulse blaster synchronizes the timing of the laser, RF source, and camera. (d) Timing sequence for data acquisition, controlled by the pulse blaster. The laser, camera, and RF source are switched on and off in a cyclic manner. Each cycle corresponds to a specific RF excitation, ensuring precise synchronization between optical and RF excitation during optically detected magnetic resonance (ODMR) measurements.

The negatively charged NV center in diamond (Figure 3a) is a spin‐1 quantum system, whose electronic ground state is a triplet configuration with spin sublevels *m*_s_ = 0 and *m*_s_ = ±1, as shown in Figure 3b. The zero‐field splitting between the *m*_s_ = 0 and *m*_s_ = ±1 states is *D*_gs_ = 2.87 GHz, which corresponds to a resonant transition that can be coherently driven by a microwave field. Moreover, under optical excitation, the NV center exhibits spin‐dependent fluorescence, where the *m*_s_ = 0 state yields stronger emission than the *m*_s_ = ±1 states. This fluorescence contrast arises from spin‐selective intersystem crossing into a non‐radiative singlet pathway, resulting in approximately a 20% reduction in the photon emission rate when the NV center is in the *m*_s_ = ±1 states [38]. It is these unique optical and spin properties that make the combination of super‐resolution microscopy and ODMR spectrometry possible.

A custom‐built optical system integrating the aforementioned SIM‐based super‐resolution scheme with ODMR spectrometry is shown in Figure 3c. This integrated setup enables the simultaneous acquisition of high‐resolution fluorescence images and spin‐dependent fluorescence contrast, thereby providing comprehensive insight into the photophysical and spin properties of NV centers embedded within the diamond. A continuous‐wave 532‐nm laser is modulated and delivered through a mechanically deformed multimode optical fiber to generate dynamic speckle illumination, which is crucial for SIM. Consistent with the earlier description, these speckle patterns are transformed, upon entering the high‐refractive‐index diamond, into high‐*k* excitation fields capable of addressing NV centers with sub‐diffraction‐limited spatial modulation. Simultaneously, a synchronized radio‐frequency (RF) electromagnetic field is applied through a loop antenna to induce spin transitions (see Methods for details).

As illustrated in Figure 3d, a digital pulse generator precisely coordinates the timing for data acquisition. For each static speckle illumination pattern, fluorescence images are sequentially acquired across a full RF frequency sweep under both RF‐on and RF‐off conditions, with the emission collected through a 600‐nm long‐pass filter and recorded by a scientific CMOS (sCMOS) camera. This time‐multiplexed acquisition enables the extraction of spin‐dependent fluorescence contrast under spatially varying excitation conditions, forming the basis for speckle‐assisted ODMR imaging. After completing data acquisition for one speckle pattern, the fiber is mechanically stretched again to generate a new illumination condition, and the process is repeated (see Methods for details).

**FIGURE 4:**
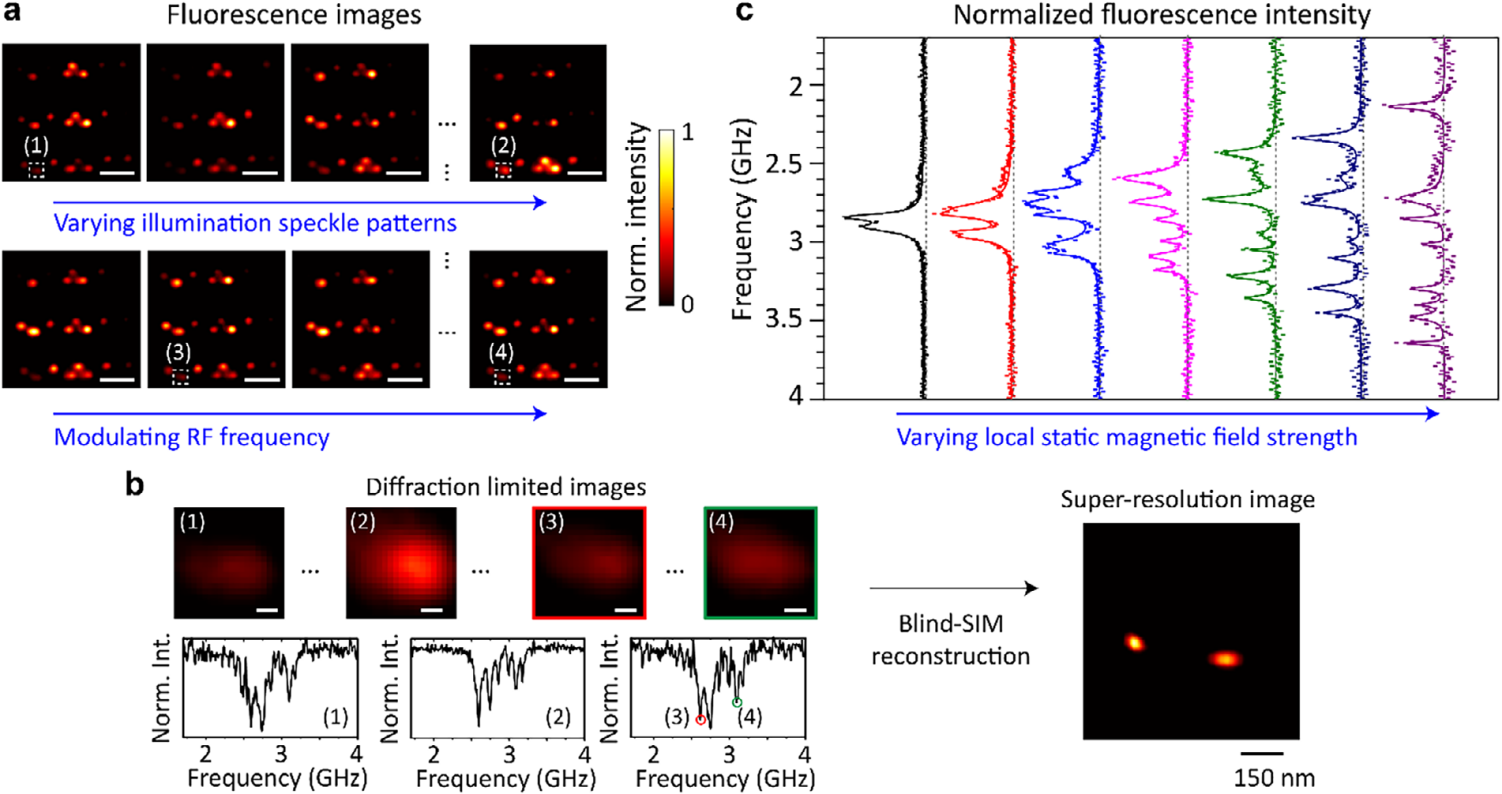
Results of super‐resolution imaging and ODMR contrast mapping. (a) Fluorescence images of NV centers acquired under varying laser speckle patterns (top panel) and modulated RF (microwave) frequencies (bottom panel). Each image corresponds to a different combination of spatial excitation and spin resonance conditions, contributing to structured illumination. Scale bars: 2.5 µm. (b) Diffraction‐limited images (top panel) and their corresponding ODMR spectra (bottom panel) for representative spatial locations labeled (1)–(4). A blind‐SIM reconstruction algorithm applied to the full dataset yields a super‐resolution image of spatially close NV centers. Scale bars: 150 nm. (c) Normalized fluorescence intensity as a function of RF frequency under various local static magnetic field strengths. Therefore, the observed Zeeman splitting reflects the magnetic field strength at each NV site, enabling spin‐resolved super‐resolution imaging.

The resulting dataset comprises a series of fluorescence images acquired under varying speckle illumination patterns and RF modulation states. As shown in Figure 4a, each image frame corresponds to a unique combination of spatial excitation and spin resonance conditions, enabling the collection of a highly multiplexed dataset. Figure 4b presents diffraction‐limited images alongside their corresponding ODMR spectra at representative locations. These diffraction‐limited images are subsequently processed using a blind‐SIM reconstruction algorithm (see Methods for details) [20, 28, 39] to recover high‐resolution images of the NV centers. Furthermore, by sweeping the external electromagnetic field, frequency‐resolved fluorescence responses are obtained, as visualized in Figure 4c, where the Zeeman splitting of the ODMR peaks varies systematically with the local static magnetic field strength. The observation of eight distinct resonance peaks corresponds to the four possible crystallographic orientations of NV center axes in diamond, each comprising a pair of spin transitions. This confirms that the measured signal arises from an ensemble of NV centers with different alignments relative to the static magnetic field. Therefore, such an integrated approach—combining high‐index‐mediated speckle compression, RF‐controlled spin modulation, and computational image reconstruction—enables super‐resolution imaging with simultaneous ODMR contrast mapping.

### 2.4 Sub‐100 nm Resolution Imaging of Laser‐Patterned NV Centers

**FIGURE 5:**
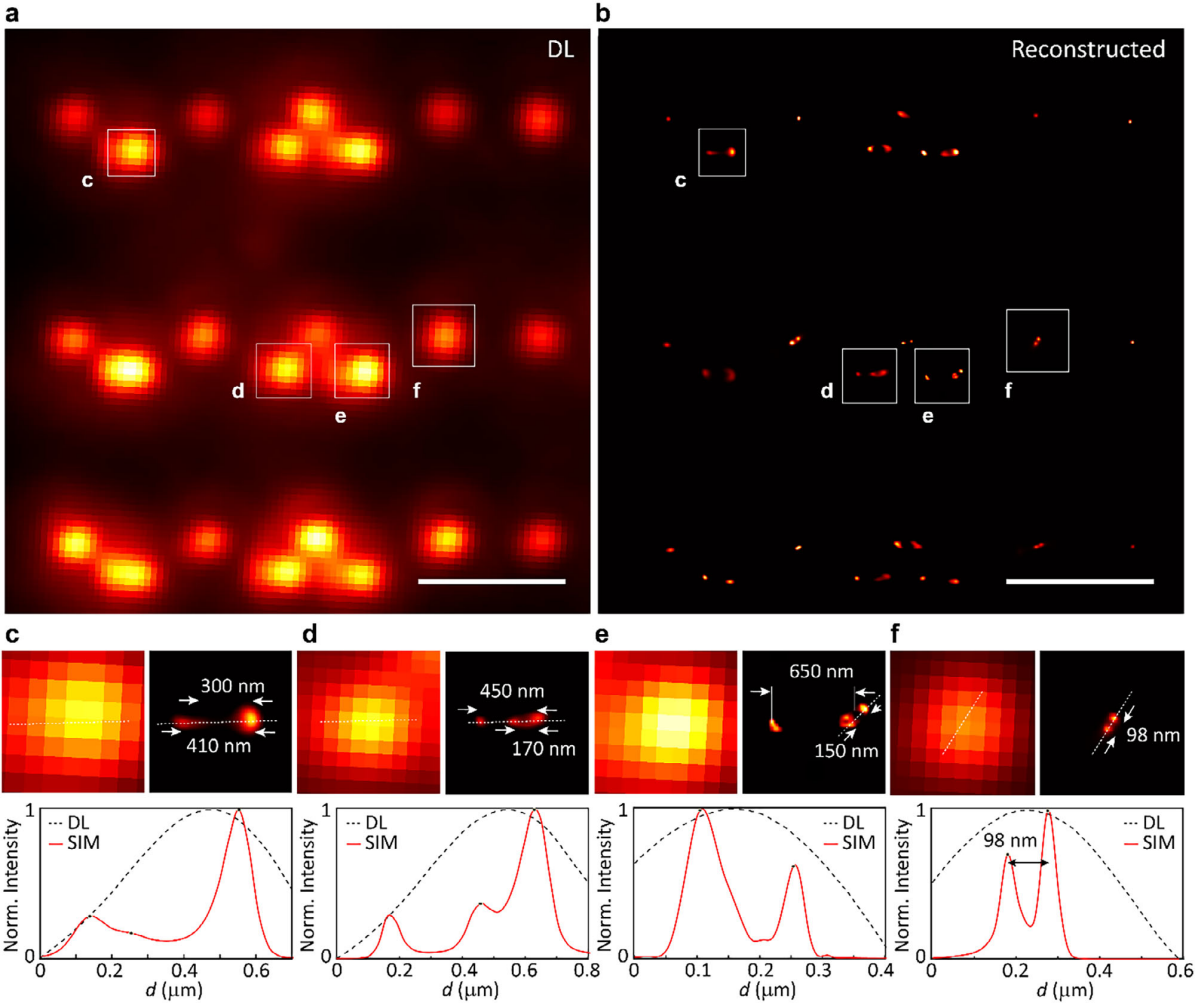
Super‐resolution imaging of laser‐written NV centers in diamond. (a) Diffraction‐limited (DL) image. (b) Corresponding reconstructed SIM image. Fluorescence images were acquired with a 60× objective lens (0.9 NA). Scale bars: 2.5 µm. (c–f) Zoomed‐in regions and corresponding line profiles from the regions labeled in (a) and (b), comparing diffraction‐limited imaging (black dashed) with SIM reconstruction (red). Features with separations down to <100 nm are resolved, demonstrating a more than twofold improvement in spatial resolution.

To assess the spatial resolution enhancement achieved by our SIM approach, laser‐written NV center arrays were imaged under both diffraction‐limited and super‐resolved conditions (Figure 5a,b). In the SIM reconstructions, discrete NV centers within each patterned region become clearly resolved. As schematically illustrated in Figure 1e, the laser‐written structures consist of pairs of 200 nm × 200 nm square dots. The center‐to‐center separations between paired squares were designed to be 250 nm, 300 nm, and 400 nm, corresponding to NV‐forming regions spanning approximately 50–450, 100–500, and 200–600 nm, respectively. It is worth noting that, although the stochastic nature of NV center generation exists within each irradiated square, the actual spacing between individual NV centers can be considerably smaller, allowing for sub‐100 nm separations even in nominally larger patterns. Therefore, the resulting ensembles exhibit variable density and spatial distribution within the patterned regions.

In the diffraction‐limited image (Figure 5a), emission from closely spaced NV ensembles appears merged, particularly when their separations are below ∼360 nm. In contrast, the SIM reconstructed image (Figure 5b) resolves these features into distinct spots, closely reproducing the intended pattern geometry. Zoomed‐in regions and corresponding line profiles from four representative areas labeled “c”–“f” are presented in Figure 5c–f. For instance, in region “c”, two emission peaks separated by ∼300 nm are indistinguishable in the diffraction‐limited image but are clearly resolved in the SIM reconstruction. In region “d”, features separated by ∼170 nm are successfully resolved, while region “e” demonstrates resolution of features separated by as little as ∼150 nm. Most notably, in region “f”, two features separated by only ∼98 nm are distinctly resolved, demonstrating sub‐100 nm resolution and representing a more than twofold improvement compared to the diffraction limit. Because the laser‐written NV layer resides approximately 100 µm beneath the diamond surface, direct nanoscale ground‐truth characterization of the buried emitter distribution (e.g., via scanning electron microscopy or atomic force microscopy) is technically prohibitive. The resolution estimates in bulk diamond are therefore based on the optically resolved separations revealed by the SIM reconstruction. The fidelity of our SIM reconstruction is further supported by comparison with ground‐truth images obtained from nanodiamond measurements, where emitter positions are independently confirmed by scanning electron microscopy (see Figure S3), and by the observation that the same super‐resolved emitters exhibit characteristic ODMR contrast under microwave excitation, identifying them as genuine NV ensembles rather than spurious optical artifacts.

### 2.5 Spatially Resolved Magnetic Imaging Via Laser‐Patterned NV Centers

**FIGURE 6:**
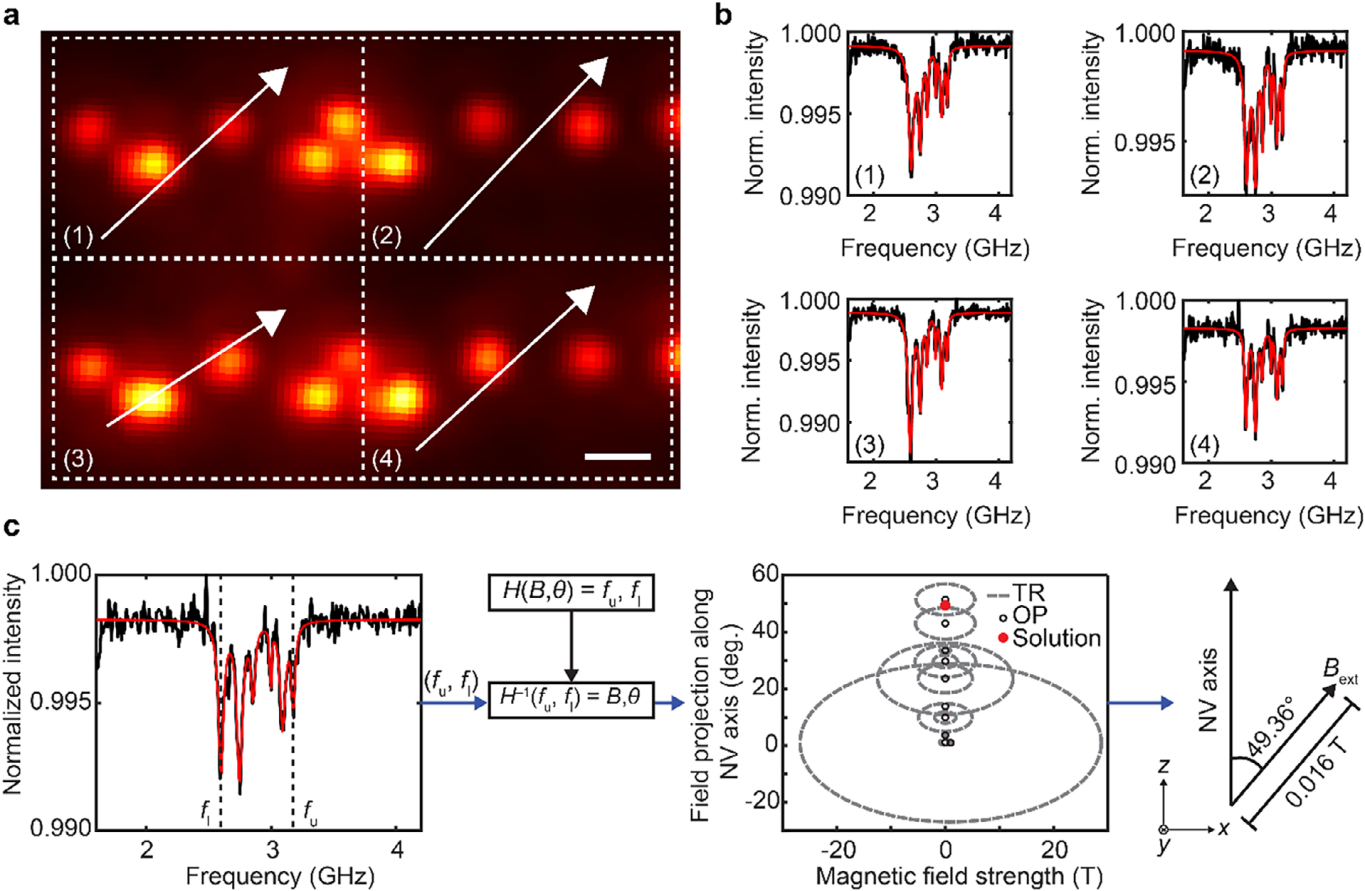
Vector magnetic field retrieval from widefield ODMR images of laser‐written NV centers. (a) Widefield fluorescence image of laser‐patterned NV centers, segmented into four tiles for local static magnetic field analysis. White arrows indicate the calculated magnetic field vector in each tile. Scale bar: 1 µm. (b) ODMR spectra extracted from the corresponding four tiles in (a). Black curves represent experimental data, while red lines are Lorentzian fits. (c) Representative ODMR spectrum showing multiple resonance dips used to extract peak frequencies *f*_l_ and *f*_u_, corresponding to the Zeeman splitting. The inverse problem is solved using the NV center Hamiltonian to estimate the magnetic field strength and its projection angle relative to the NV axis. The retrieved magnetic field vector (red dot) is determined via optimum path (OP) within a trusted radius (TR) in the field parameter space.

Whereas Figure 5 demonstrates the capability of the imaging system to achieve super‐resolved visualization of NV center ensembles, Figure 6 highlights an additional functionality enabled by the integration of ODMR spectrometry. By analyzing the spin‐dependent fluorescence response under varying RF electromagnetic field conditions, the system provides spatially resolved maps of local static magnetic field vectors [40, 41, 42, 43]. This dual capability to extract both nanoscale structural details and quantitative magnetic field information within a single optical platform underscores the versatility and significance of the proposed method.

To perform the local static magnetic field imaging, the widefield image was divided into multiple grid regions, as shown in Figure 6a. For each grid, pixel intensities were tracked across the sequentially acquired image stack to reconstruct the corresponding ODMR spectrum, as presented in Figure 6b. The observed ODMR signal consists of six peaks, representing magnetic hyperfine splitting arising from three crystallographically distinct NV center orientations. The hyperfine splitting is proportional to the projection of the magnetic field along each NV center axis. For further analysis, we focused on the outermost ODMR peaks, which exhibit the maximum splitting and are assumed to originate from NV centers whose axes are closely aligned with the direction of the magnetic field. This is defined as the *z*‐axis in the analysis, and the magnetic field projection along this axis was calculated by solving the NV center Hamiltonian (Figure 6c; see Methods). The calculated magnetic field is shown in Figure 6a, overlaid on the widefield image. The calculations resulted in uniform magnetic field vector components across the widefield area, as expected, since the magnetic field lines from the nearby magnet are uniformly distributed over a range of several tens of millimeters in our experiments.

While the underlying ODMR‐based vector reconstruction is based on well‐established physical principles, the current implementation serves as a proof‐of‐concept for integrating super‐resolution with magnetometry. Future optimizations focusing on advanced readout schemes, such as lock‐in based camera detection, could further enhance the magnetic sensitivity and precision of this platform.

To rigorously assess the contribution of spin‐dependent modulation, we reconstructed images using only speckle illumination (at a fixed, off‐resonant microwave frequency) using the same number of frames as in the full dual‐modulation dataset (see Figure S4). The dual‐modulation (speckle + ODMR) reconstruction exhibits more robust separation of closely spaced emitters, confirming that ODMR‐induced modulation improves reconstruction stability under identical sampling conditions.

The synergy between blind‐SIM and ODMR modulation bridges a critical gap in subsurface quantum sensing. By surmounting the diffraction limit, this platform enables the study of physical phenomena previously obscured by spatial averaging. As demonstrated, this high‐resolution capability is essential for applications such as resolving closely spaced spin clusters, imaging nanoscale magnetic domain walls, and probing localized strain fields deep within the diamond bulk. Such mapping provides the necessary spatial precision to correlate local quantum signals with their nanometric physical sources, which is inaccessible with existing widefield ODMR techniques alone.

Importantly, to further evaluate the reliability of our vector magnetic‐field reconstruction, we performed a quantitative uncertainty analysis by accounting for experimental noise propagation through the nonlinear Hamiltonian inversion. By statistically analyzing multiple independent measurements across various laser‐written NV center sites, we found that the relative uncertainty in magnetic field strength remains below ∼6%, while the angular precision is maintained within 1–3°. These results confirm that the reconstruction process is robust against experimental ODMR noise and exhibits high numerical stability (see Figure S5).

## 3 Discussion

Although localization‐based super‐resolution techniques can, in principle, offer comparable or even higher nominal resolution, they typically necessitate sparse, independently blinking single emitters and are therefore not naturally suited for imaging subsurface, laser‐written NV ensembles in bulk diamond. In contrast, the speckle‐based structured illumination approach demonstrated here is intrinsically compatible with densely distributed NV centers and seamlessly integrates with wide‐field ODMR acquisition, providing a practical framework for multimodal quantum imaging in this configuration. In this setting, the method not only resolves sub‐100‐nm separations between NV ensembles but also enables the simultaneous retrieval of ODMR spectra and magnetic‐field information across multiple regions. Furthermore, SIM operates at comparatively moderate excitation intensities, which helps preserve NV spin coherence and minimizes laser‐induced heating during bulk‐diamond ODMR measurements. Taken together, these features indicate that structured illumination microscopy is not redundant relative to localization‐based approaches, but instead offers a practically advantageous and conceptually coherent route to multimodal wide‐field imaging of NV centers in diamond.

In the present work, the high‐*k* excitation components are accessed indirectly through their modulation effects encoded within the wide‐field fluorescence images and the subsequent reconstruction process. While a direct mapping of the compressed speckle field at the diamond interface—for example, via near‐field scanning optical probes or a thin fluorescent probe layer imaged from the high‐index side—would provide complementary validation of the high‐frequency excitation structure, such efforts remain an interesting direction for future work.

While imaging at a depth of ∼100 µm inside a high‐refractive‐index diamond with a 0.9‐NA air objective inevitably introduces spherical aberrations, the use of a blind‐SIM reconstruction scheme—which jointly estimates the effective illumination patterns and the sample distribution—and the additional ODMR‐based fluorescence modulation together help maintain robust super‐resolved imaging performance under these aberrated conditions. Looking forward, the integration of hardware‐level corrections, such as adaptive optics or immersion objectives, could further mitigate these spherical aberrations and enable even higher‐fidelity imaging.

## 4 Conclusion

We have presented an advanced imaging strategy that leverages the high refractive index of diamond to push the spatial resolution limits of SIM for NV center imaging. Specifically, the intrinsic optical properties of diamond are utilized to compress incoming low‐spatial‐frequency speckle patterns into sub‐diffraction excitation features, thereby effectively extending the accessible spatial‐frequency range for super‐resolution reconstruction. Furthermore, by integrating this optical advantage with ODMR measurements, we have realized a multimodal imaging platform capable of resolving NV center arrays with enhanced spatial precision while simultaneously detecting their spin‐dependent fluorescence responses. It is worth noting that the RF frequency‐dependent modulation of fluorescence inherent to ODMR serves as a complementary structured illumination mechanism, reinforcing the super‐resolution reconstruction process. Because the laser‐written NV layer resides beneath the diamond surface, direct nanoscale ground‐truth characterization of the buried emitter distribution is not readily accessible; accordingly, the sub‐100‐nm separations and vector‐field maps are presented as optical‐ and ODMR‐constrained estimates based on reconstructed peak separations and Hamiltonian‐based inversion of measured ODMR resonances. Looking ahead, the subsurface patterning of NV centers within bulk diamond opens avenues for 3D quantum architectures, long‐term biocompatible sensing, and advanced materials diagnostics. This study establishes a foundation for future developments that combine intrinsic material properties with computational super‐resolution techniques, paving the way for expanded functionality in NV center‐based imaging and quantum sensing applications.

## 5 Methods

### 5.1 Experimental Setup

A 532 nm laser diode (Changchun New Industries Optoelectronics Tech. Co., Ltd.) with an output power of approximately 160 mW was modulated using an acousto‐optical modulator (AOM, ISOMET) to produce a first‐order diffracted beam, which was subsequently coupled into a multimode optical fiber (core/cladding diameter: 62.5/125 µm). By mechanically stretching the optical fiber using a stepping motor and applying a motorized fan at the output end, a series of spatially diverse speckle patterns was generated at specific time intervals. These patterns were projected onto the diamond sample through an excitation filter (512–536 nm) and a high‐NA objective (60×, 0.9 NA), with modulation occurring over time due to both mechanical deformation of the fiber and refractive index changes at the diamond interface. To achieve dynamic control over fluorescence emission, an RF source (Holzworth Instrumentation, HSM4001B) was applied simultaneously. The RF signal was routed through a switch (Mini‐Circuits, ZFSWA2‐63DR+) and amplified (Mini‐Circuits, ZHL‐1W‐63‐S+), then delivered to an RF antenna positioned near the diamond. The RF frequency was modulated with a step size of 10 MHz over a specified range to induce ODMR, resulting in spin‐dependent variations in fluorescence intensity. These changes in brightness effectively enhanced the spatial contrast of the speckle illumination. A programmable digital pulse sequence generator (Spincore Pulseblaster PBESR‐PRO‐500‐USB‐RM2) synchronizes the operation of the laser, RF source, and camera at a clock rate of 500 MHz. For continuous‐wave ODMR analysis, the laser was kept on, while the camera recorded images during alternating on‐off states of the RF electromagnetic field. The resulting fluorescence emission was collected through a dichroic mirror (cut‐off: 550 nm), an emission filter (600 nm long pass), and a sCMOS camera. For each static speckle pattern, a full RF frequency sweep was performed, allowing the collection of image sets containing spin‐modulated fluorescence. After completing data acquisition for each speckle pattern, the fiber was stretched again to generate a new illumination condition, and the process was repeated. The optical characteristics of the NV centers in diamond were double‐checked and investigated in more detail using a MicroTime 200 time‐resolved confocal fluorescence microscope (PicoQuant, NFEC‐2025‐04‐305233).

### 5.2 SIM Super‐Resolution Image Reconstruction

All image reconstruction procedures were conducted using MATLAB. The blind‐SIM [39] method reconstructs high‐resolution images by applying an iterative reconstruction algorithm to a series of low‐resolution images generated under random speckle illumination. The algorithm minimizes a cost function iteratively, quantifying the discrepancy between the measured and predicted image data, and progressively refines the estimates to obtain a super‐resolved image. A total of 31,320 raw images were acquired to accommodate the requirements of ODMR RF scanning and signal averaging; however, only 100–300 frames from this stack were required for the blind‐SIM reconstruction. The camera frame period, including readout, was 50 ms (20 frames/s) at an excitation power of ∼60 mW. Accordingly, acquisition of the full 31,320‐frame dataset corresponds to ∼1,566 s (∼26.1 min, excluding minor hardware overhead). The 31,320 frames decompose as 261 RF frequency points × 20 frames per point (10 RF‐on + 10 RF‐off) × 6 repetitions for averaging. This indicates that acquisition times could, in principle, be substantially reduced by optimizing the number of speckle realizations and RF sampling points for applications requiring higher temporal resolution. In particular, once the ODMR resonance frequencies are identified, subsequent imaging can operate by toggling the RF at (or near) selected resonance frequencies without a full 261‐point scan; in this reduced protocol, 100–300 frames correspond to ∼5–15 s at the current frame period. Acquisition time can be further reduced by increasing the excitation laser power to shorten the required exposure time. Future implementations combining lock‐in detection and neural‐network‐assisted reconstruction are expected to further improve the temporal resolution of this subsurface imaging platform. The super‐resolution reconstruction code [20] was executed on a GPU‐accelerated system featuring an NVIDIA GeForce RTX 5090 and a 44‐core CPU for an image size of 300 × 300 pixels.

### 5.3 ODMR Fitting of the Vector Magnetic Field

The Hamiltonian describing the ODMR process of an NV center in diamond depends on the local static magnetic field and its projection along the NV center axis. The characteristic equation for this Hamiltonian is a third‐order polynomial, with its roots corresponding to the frequency eigenvalues. By extracting the frequency eigenvalues *f*_u_ and *f*_l_ from the experimental ODMR data, the characteristic equation is iteratively solved to calculate the vector magnetic field. The back‐calculation is performed by substituting the frequency eigenvalues into the NV center Hamiltonian. Then, a numerical solver in MATLAB calculates eigenfrequencies and matches those obtained experimentally to minimize the search area as identified in Figure 6c. The process will continue until the difference between experimental and theoretical eigenfrequency pairs converges within machine precision. To improve robustness and mitigate the influence of model and fitting uncertainties, multiple ODMR transitions from different NV center orientations are simultaneously fitted at each spatial region, providing an overdetermined set of constraints for the magnetic‐field vector. Particular emphasis is placed on the outermost resonances, which are associated with NV centers whose axes are most closely aligned with the external magnetic field. Because these extrema exhibit the largest Zeeman splitting and reduced peak overlap among different NV center orientations, they offer a more reliable constraint for field retrieval and help reduce degeneracy and ambiguity in the reconstruction. A least‐squares fitting procedure with outlier rejection is employed, and spatial regions with insufficient signal‐to‐noise ratio are excluded from the final magnetic‐field map.

## Conflicts of Interest

The authors declare no conflict of interest.

## Supporting information




**Supporting File**: advs74340‐sup‐0001‐SuppMat.docx.

## Data Availability

The data that support the findings of this study are available from the corresponding author upon reasonable request.
