# Supplementary material for: Multimodal Super‐Resolution Imaging of Nitrogen‐Vacancy Centers via High‐Index‐Induced Structured Illumination Microscopy and Optically Detected Magnetic Resonance Spectrometry
Source: Adv Sci (Weinh). 2026 Mar 3;13(24):e22495. doi: 10.1002/advs.202522495 (PMC13115950; doi:10.1002/advs.202522495)
Supplement: Supplementary file 1 — Supporting File: advs74340‐sup‐0001‐SuppMat.docx. [file ADVS-13-e22495-s001.docx]

Supporting Information

Multimodal Super-Resolution Imaging of Nitrogen-Vacancy Centers via High-Index-Induced Structured Illumination Microscopy and Optically Detected Magnetic Resonance Spectrometry

Kyu Ri Choi^‡^, Mohammed Zia Jalaludeen^‡^, Samuel Begumya, Yan Qiu Du, Dong Hee Park, Bin Chan Joo, Jae Hwan Yoo, Síle Nic Chormaic, Shilong Li^*^, Yeon Ui Lee^*^

^‡^These authors contributed equally to this work.

^*^[shilong.li@zju.edu.cn](mailto:shilong.li@zju.edu.cn), [yeonuilee@cbnu.ac.kr](mailto:yeonuilee@cbnu.ac.kr)

**Contents**

**S1. Bright-field imaging of the interface layer in bulk diamond**

**S2. Formation of NV centers by femtosecond laser irradiation**

**S3. SIM super-resolution imaging of nanodiamond particles**

**S4. Control comparison of speckle-only and dual-modulation reconstructions**

**S5. Quantitative uncertainty analysis of vector magnetic field reconstruction**

**S1. Bright-field imaging of the interface layer in bulk diamond**

The surface, interface, and bottom regions of the bulk diamond were examined using wide-field bright-field microscopy. As shown in **Figure S1b**, a distinct interface layer located approximately 100 μm beneath the surface is optically identifiable due to contrast variation and focus. In comparison, the surface (Figure S1a) and bottom regions (Figure S1c) exhibit a relatively uniform contrast with no clearly defined structural features. The presence of a focusable layer within the bulk diamond suggests a localized change in the material’s microstructure or optical properties at this interface depth.


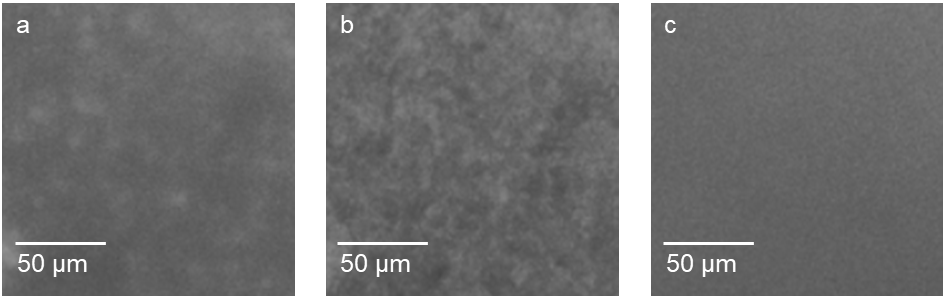


**Figure S1.** Bright-field imaging of bulk diamond. Bright-field microscopy images of the bulk diamond sample are shown at various depths: (a) the surface, (b) the interface layer (approximately 100 μm below the surface), and (c) the bottom.

**S2. Formation of NV centers by femtosecond laser irradiation**

In this work, we employed the femtosecond laser writing method to directly generate NV centers in diamond, eliminating the need for thermal annealing. This simple approach results in high-density NV ensembles with precise spatial control and minimal post-processing.

The traditional method of forming NV centers by high-energy electron or ion irradiation followed by high-temperature annealing (≥800°C) enables vacancy creation and subsequent migration to substitutional nitrogen atoms, but requires complex infrastructure and introduces unwanted damage or contamination^[31]^. Femtosecond laser irradiation is a promising alternative due to the non-invasive nature of this approach and its three-dimensional spatial control ability^[32–36]^. It has been proposed that laser-induced photogenerated neutral vacancies (GR1 centers) recombine with substitutional nitrogen atoms near the focal volume, forming NV centers^[36]^. Additionally, localized heating from laser pulses can act as a point heat source, diffusing vacancies and thus eliminating the need for external furnace annealing^[36]^.

The NV centers in our experiment were created using a Zeiss LSM 710 confocal microscope equipped with a tunable infrared femtosecond pulsed laser (Coherent Chameleon Ultra II) operating between 690 nm and 1064 nm. The presence of NV centers was confirmed in situ using the same confocal microscope by examining their characteristic spectra. The successfully fabricated NV centers were observed exclusively within an interface layer located approximately 100 µm beneath the diamond surface.

To evaluate the microscope system’s positioning precision, a statistical analysis on a large-scale calibration array was performed (see **Figure S2**). The results indicate a lateral positioning uncertainty of approximately 240 nm. This suggests that the slight deviations and multi-peak features observed in the reconstructed dot arrays (Figure 5) primarily stem from the intrinsic stochastic formation of multiple NV centers and their varied crystallographic orientations, rather than reconstruction instability.


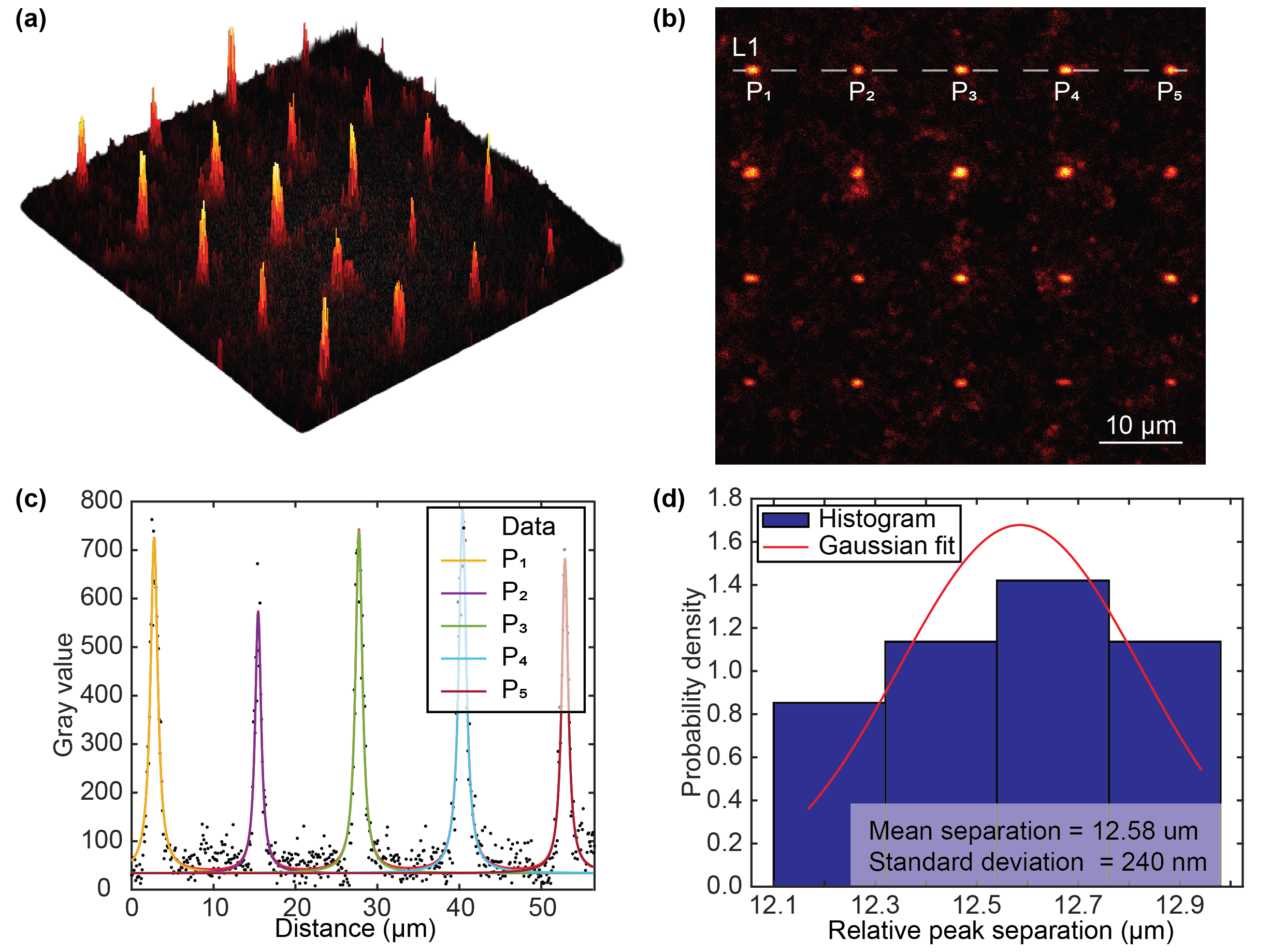


**Figure S2.** Statistical analysis of positioning uncertainty for laser-written NV centers. (a,b) Fluorescence images of laser-written NV center arrays used for positioning uncertainty estimation. (c,d) Corresponding statistical results of the measured peak positions (c) and the resulting peak-to-peak separations (d).

**S3. SIM super-resolution imaging of nanodiamond particles**

Since the laser-written NV layer is embedded within the bulk diamond, direct characterization methods such as scanning electron microscopy (SEM) cannot be applied to the NV layer. As a result, the exact dimensions and spacing of the inscribed features cannot be measured directly. To address this limitation, we evaluated the performance of the blind-SIM reconstruction algorithm using a reference sample for which ground truth data are available. Specifically, blind-SIM reconstruction was applied to NV nanodiamond particles on a glass substrate. This comparative approach provides a reliable means of assessing the resolution enhancement and reconstruction accuracy of the algorithm.

The SIM reconstruction was conducted on approximately 70 nm NV nanodiamond particles spin-coated on a cover glass substrate. An SEM image of the sample is shown in **Figure S3a**, revealing adjacent NV nanodiamond particles that appear to be diffraction-limited in wide-field fluorescence microscopy (Figure S3b). After blind-SIM reconstruction, two distinct nanodiamond particles were clearly resolved (Figure S3c). The overlaid image (Figure S3d) shows strong spatial correlation between the SIM results and the SEM ground truth, validating the resolution performance of the reconstruction process.


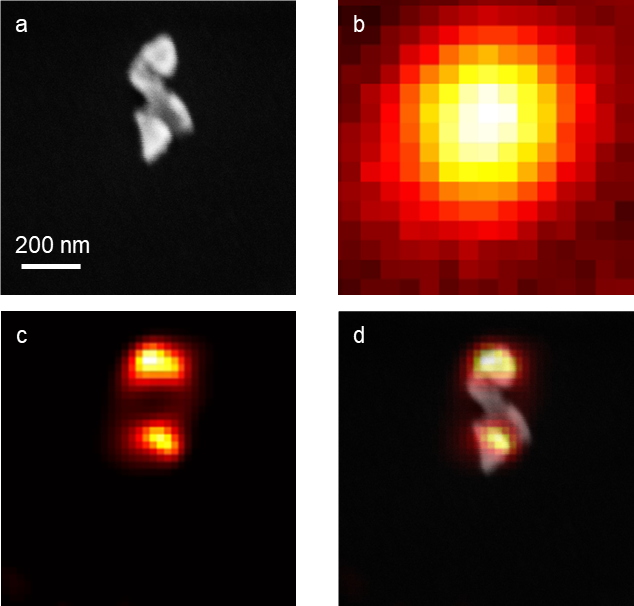


**Figure S3.** SIM super-resolution imaging of nanodiamond particles. SEM image of nanodiamond particles (a), diffraction-limited image of nanodiamond sample spot (b), SIM-resolved image of the sample spot (c), and overlay of the super-resolved image and the SEM image of the sample spot (d).

**S4. Control comparison of speckle-only and dual-modulation reconstructions**

To quantify the contribution of ODMR-induced modulation to the reconstruction quality, we performed a control analysis using two datasets with an identical sampling budget (100 frames each). In the first case, 100 frames were randomly selected from a sequence acquired under varying speckle illumination with the microwave frequency fixed far off-resonance, so that no appreciable spin-dependent fluorescence modulation was present (speckle-only condition). A blind-SIM reconstruction using this speckle-only dataset (**Figure S4a**) already surpasses the diffraction limit; however, the reconstructed emitters exhibit broadened peaks, reduced peak-to-background ratio, and noticeable residual artifacts in regions containing densely packed NV ensembles.

In the second case, another set of 100 frames was selected from the full dataset that includes the ODMR frequency sweep, so that both spatial speckle modulation and spin-resonant fluorescence modulation are present (dual-modulation condition). When these 100 dual-modulation frames are used for blind-SIM reconstruction (Figure S4b), the resulting image shows sharper and more compact emission peaks, deeper intensity dips between closely spaced features, and a cleaner background compared to the speckle-only case.

These observations support the conclusion that microwave-driven ODMR contrast acts as a complementary structured excitation in the temporal/spectral domain. This additional modulation improves the conditioning of the inverse problem, enhancing super-resolution reconstruction quality and stability beyond what speckle-only SIM can achieve with the same number of frames.


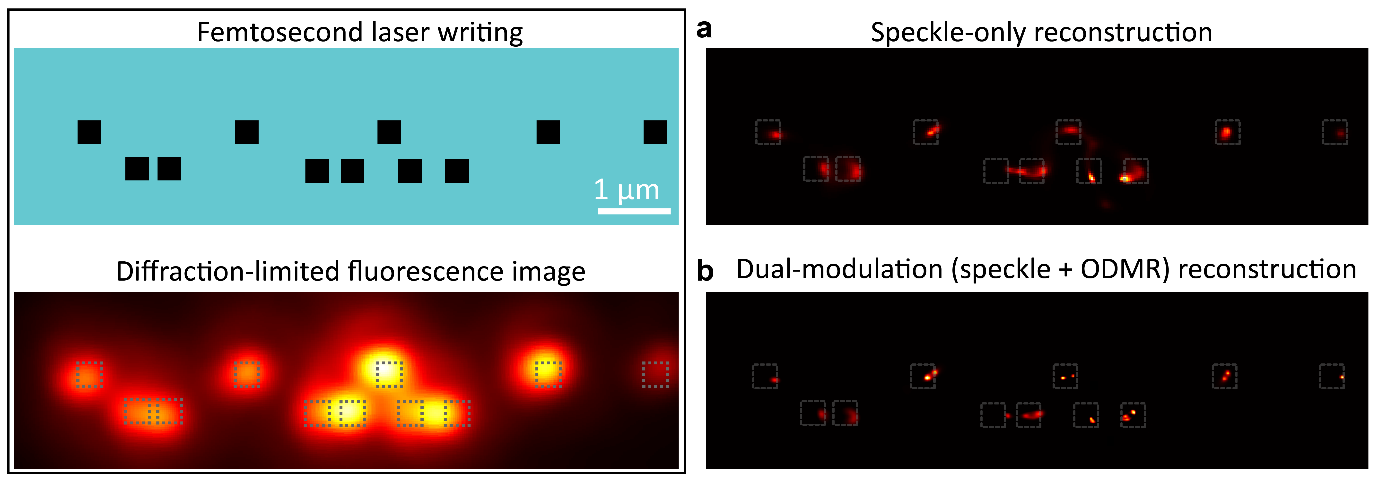


**Figure S4.** Control comparison of speckle-only and dual-modulation (speckle + ODMR) reconstructions. Top left: Schematic of the laser-written NV-ensemble pattern. Bottom left: Corresponding diffraction-limited fluorescence image acquired under wide-field excitation. (a) Super-resolution image reconstructed from 100 frames acquired with speckle-only modulation, where the microwave frequency is kept off-resonance (no ODMR-induced fluorescence modulation). While several emitters are resolved, residual background and reconstruction artifacts remain within the dashed regions. (b) Super-resolution image reconstructed from 100 frames acquired with dual-modulation (speckle + ODMR frequency sweep). Here, the ODMR-induced, spin-dependent fluorescence modulation provides an additional contrast channel. For the same number of frames, the dual-modulation reconstruction yields cleaner emitter separation, reduced background, and improved localization stability, illustrating that ODMR modulation enhances the robustness and fidelity of the SIM reconstruction beyond speckle patterns alone.

**S5. Quantitative uncertainty analysis of vector magnetic field reconstruction**

To evaluate the reliability of the vector magnetic field reconstruction and quantify how experimental noise propagates through the nonlinear Hamiltonian inversion process, we performed a systematic uncertainty analysis.

We selected five distinct laser-written NV center sites (i.e., *P*_1_–*P*_5_), as indicated in **Figure S5a**. For each site, the ODMR spectrum was recorded six times under identical experimental conditions to capture the inherent measurement noise. Instead of fitting the mean spectrum directly, the Hamiltonian inversion algorithm was applied independently to each of the six raw ODMR curves. This allowed us to generate a statistical distribution of the reconstructed magnetic field parameters (magnitude *B* and angle $\theta$).

Figure S5b illustrates a representative ODMR dataset for site *P*_1_, where the shaded area represents the experimental variance across the six measurements. The resulting distributions for the magnetic field vector components are shown in Figure S5c, with the corresponding standard deviations summarized in Figure S5d to visualize the reconstruction stability for this specific site. To evaluate the spatial consistency of this performance, we extended the same analysis to all five NV center sites. As summarized in **Table S1**, the relative standard deviation for the magnetic field strength across all five sites remains consistently below ~6%, with angular uncertainty typically within the range of 1–3°. The high consistency of these uncertainties across spatially separated NV centers, despite local variations in fluorescence intensity, demonstrates that: (i) The reconstruction error is dominated by the signal-to-noise ratio of the ODMR signal rather than numerical instabilities in the algorithm; and (ii) the nonlinear inversion process is robust and yields reproducible results for vector magnetometry at the nanoscale.


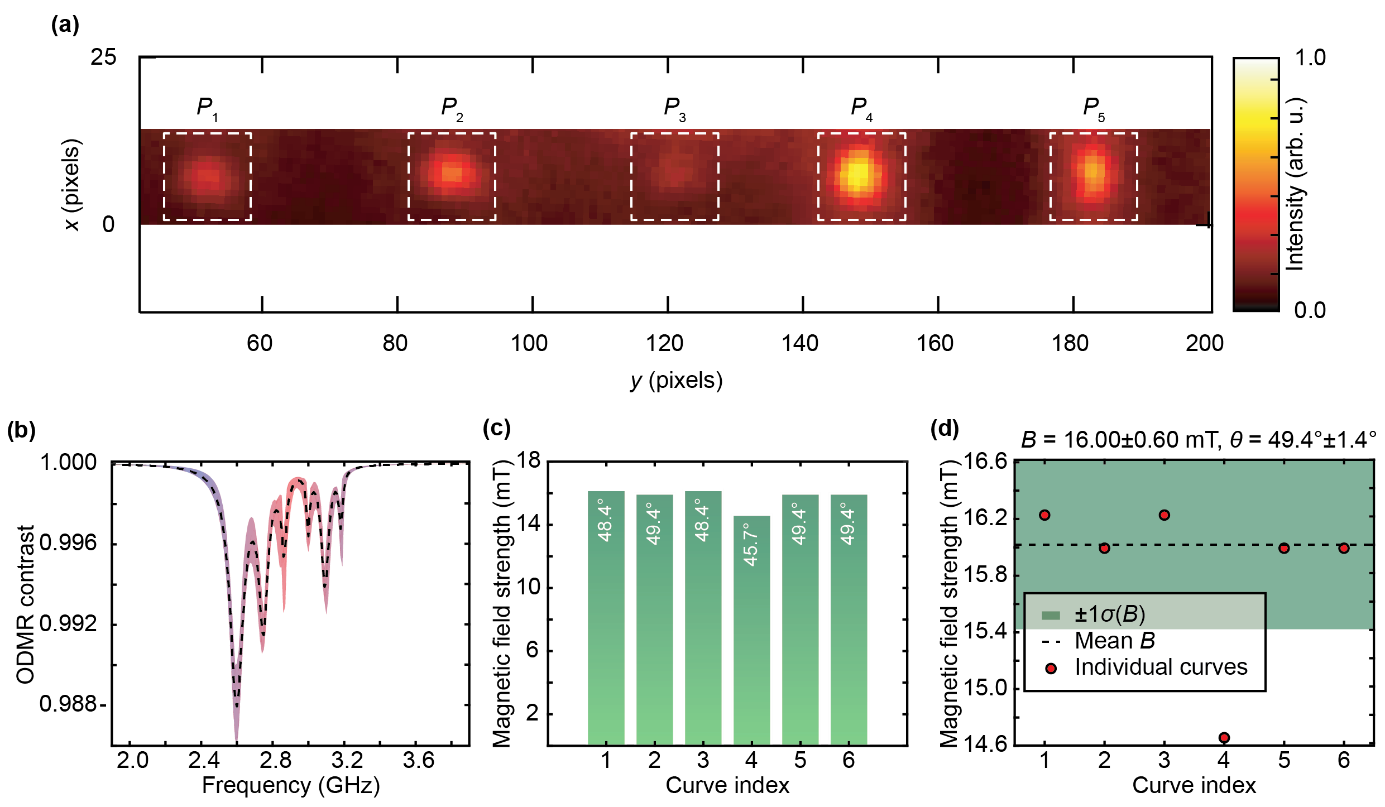


**Figure S5.** Quantitative analysis of uncertainty propagation in vector magnetic field reconstruction. (a) Fluorescence image of five representative laser-written NV center sites (*P*_1_–*P*_5_) used for statistical analysis. (b) Experimental ODMR spectrum for site *P*_1_. The dashed line represents the mean of six independent measurements, while the shaded area indicates the experimental spread. (c) Distribution of the reconstructed magnetic field parameters (magnitude *B* and angle $\theta$) derived from the six individual ODMR curves for site *P*_1_. (d) Standard deviation of the reconstructed parameters relative to the mean values, demonstrating the stability of the nonlinear Hamiltonian inversion against experimental noise.

**Table S1.** Quantification of reconstruction uncertainties and relative errors for five spatially distinct NV center sites.

| Sites | *B* (mT) | $\theta$ |
| --- | --- | --- |
| *P*_1_ | $16.00\pm0.60$ | $49.36^{\circ}\pm1.4^{\circ}$ |
| *P*_2_ | $16.00\pm0.67$ | $49.36^{\circ}\pm2.6^{\circ}$ |
| *P*_3_ | $16.00\pm0.94$ | $49.36^{\circ}\pm3.2^{\circ}$ |
| *P*_4_ | $14.90\pm0.75$ | $44.70^{\circ}\pm3.1^{\circ}$ |
| *P*_5_ | $16.00\pm0.59$ | $49.36^{\circ}\pm1.7^{\circ}$ |
